# Spatial and temporal variation at major histocompatibility complex class IIB genes in the endangered Blakiston’s fish owl

**DOI:** 10.1186/s40851-015-0013-4

**Published:** 2015-03-25

**Authors:** Tetsuo I Kohyama, Keita Omote, Chizuko Nishida, Takeshi Takenaka, Keisuke Saito, Satoshi Fujimoto, Ryuichi Masuda

**Affiliations:** Department of Natural History Sciences, Faculty of Science, Hokkaido University, Sapporo, 060-0810 Japan; Department of Natural History Sciences, Graduate School of Science, Hokkaido University, Sapporo, 060-0810 Japan; FILIN, Hachiken 2 Jo Nishi 2, Nishi-ku, Sapporo 063-0842 Japan; Institute for Raptor Biomedicine, Kushiro, 084-0922 Japan; Kushiro Zoo, Kushiro, 085-0201 Japan

**Keywords:** Balancing selection, *Bubo blakistoni*, Genetic drift, MHC class IIβ, Pyrosequencing

## Abstract

**Introduction:**

Quantifying intraspecific genetic variation in functionally important genes, such as those of the major histocompatibility complex (MHC), is important in the establishment of conservation plans for endangered species. The MHC genes play a crucial role in the vertebrate immune system and generally show high levels of diversity, which is likely due to pathogen-driven balancing selection. The endangered Blakiston’s fish owl (*Bubo blakistoni*) has suffered marked population declines on Hokkaido Island, Japan, during the past several decades due to human-induced habitat loss and fragmentation. We investigated the spatial and temporal patterns of genetic diversity in MHC class IIβ genes in Blakiston’s fish owl, using massively parallel pyrosequencing.

**Results:**

We found that the Blakiston’s fish owl genome contains at least eight MHC class IIβ loci, indicating recent gene duplications. An analysis of sequence polymorphism provided evidence that balancing selection acted in the past. The level of MHC variation, however, was low in the current fish owl populations in Hokkaido: only 19 alleles were identified from 174 individuals. We detected considerable spatial differences in MHC diversity among the geographically isolated populations. We also detected a decline of MHC diversity in some local populations during the past decades.

**Conclusions:**

Our study demonstrated that the current spatial patterns of MHC variation in Blakiston’s fish owl populations have been shaped by loss of variation due to the decline and fragmentation of populations, and that the short-term effects of genetic drift have counteracted the long-term effects of balancing selection.

**Electronic supplementary material:**

The online version of this article (doi:10.1186/s40851-015-0013-4) contains supplementary material, which is available to authorized users.

## Introduction

**Figure 1:**
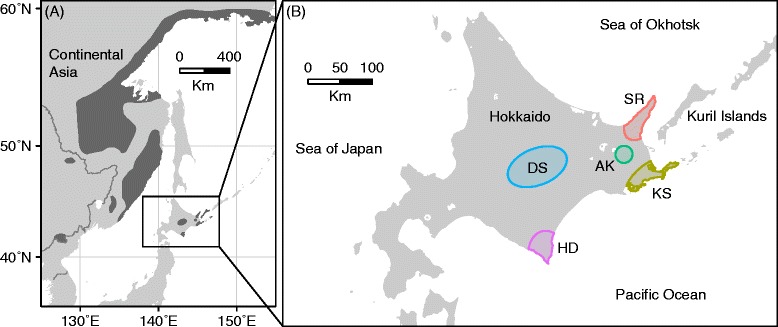
**Distribution of Blakiston’s fish owl and sampling sites of the populations investigated. (A)** Global distribution of Blakiston’s fish owl (dark area), modified from Slaght and Surmach [2]. **(B)** Sampling sites in the present study. Population abbreviations: SR, Shiretoko; KS, Konsen; AK, Akan; DS, Daisetsu; HD, Hidaka.

Blakiston’s fish owl (*Bubo blakistoni* Seebohm; hereafter ‘fish owl’), the world’s largest owl, is currently categorized as ‘Endangered’ on the IUCN Red List of Threatened Species [1]. This owl is non-migratory and endemic to northeastern Asia, comprising two subspecies: *B. b. blakistoni* in central and eastern Hokkaido and the southern Kuril islands; *B. b. doerriesi* in continental Asia, including the Russian Far East, northeastern China, and probably North Korea (Figure 1) [2]. Unlike many other owl species, the fish owl is specialized to aquatic prey, mainly freshwater fishes, and thus its habitat is limited to areas close to lakes, rivers, springs, and shoals that do not freeze in winter. In addition, it requires large, old trees with large cavities for nesting [2-4]. Over the last several decades, riparian old-growth forests, the preferred habitat for the fish owl, have been decimated due to human land use, causing a rapid decline in fish owl populations.

The current population size of the fish owl is estimated to be 120–150 individuals in Hokkaido, 70–85 in the southern Kuril Islands, and a few thousand in continental Asia [2,3,5]. The fish owl was widespread in Hokkaido until the middle of the 20th century [3,6], but its population has decreased markedly over the last several decades due to human-induced habitat loss and fragmentation, and probably fell to fewer than 100 individuals in the 1970–80s [7]. A project began in the early 1980s aimed at conservation of the fish owl, through the installation of nest boxes and artificial feeding. Thanks to these efforts, the Hokkaido population is now gradually recovering, but the risk of extinction remains high due to the loss of adaptive genetic variation and inbreeding depression in highly fragmented populations.

Recent population genetic studies using selectively neutral markers such as mitochondrial DNA (mtDNA) sequences and microsatellite genotypes revealed low overall genetic diversity in Hokkaido fish owl populations [8,9]. These studies also detected genetic differentiation among fragmented populations, indicating limited movement of owls between areas. In contrast, mtDNA sequence analyses of historical specimens, including taxidermied specimens and archaeological bones, indicated that gene flow had occurred over a broad area of Hokkaido until the middle of the 20th century [9]. Both the mtDNA and microsatellite data showed a sharp reduction in genetic diversity after around 1980 [9]. The low levels of genetic variation and genetic differentiation among subpopulations currently inhabiting Hokkaido are probably due to genetic drift and inbreeding resulting from habitat loss and fragmentation.

The major histocompatibility complex (MHC) is a multigene family that plays a crucial role in the vertebrate immune system. It contains genes coding for cell-surface glycoproteins, the MHC class I and II molecules, each of which is specifically involved in presenting antigen peptides derived from intra- or extracellular pathogens to T cells, initiating the adaptive immune response [10]. These genes are among the most polymorphic in the vertebrate genome, exhibiting high allelic diversity [11]. MHC polymorphism is generated by frequent gene duplications and deletions, intra- and inter-locus recombination or gene conversion, and the accumulation of *de novo* mutations [12,13]. In addition, the enormous variation in MHC genes is probably maintained by pathogen-driven balancing selection, mediated through either heterozygote advantage or negative frequency-dependent selection, and by sexual selection via MHC-mediated disassortative mating (reviewed in [14-17]).

Although balancing selection shapes MHC polymorphism to a great extent over the long term, MHC variation is often substantially reduced in populations that have undergone extreme bottlenecks (e.g., [18-21]). Habitat fragmentation may further accelerate the reduction in MHC diversity by strengthening the effects of genetic drift (e.g., [22-24]). Although the effects of reduced MHC diversity on the long-term viability of populations that undergo bottlenecks has remained unclear (reviewed in [25]), the example of contagious cancer in Tasmanian devils (*Sarcophilus harrisii*) strongly supports the possibility that populations with low MHC diversity are more vulnerable to outbreaks of novel infectious diseases (reviewed in [26]). Quantifying MHC diversity can thus provide an indirect measure of the immunological fitness (i.e. the potential of resistance to novel infectious diseases) of a population, and should thus be incorporated into studies of endangered species aimed at establishing conservation plans [27,28].

The first comprehensive information on the genomic structure of the MHC in birds came from studies of the domestic chicken, *Gallus gallus* [29]. The chicken MHC is more compact than the mammalian MHC; it has only two classical MHC class I and II genes, which is referred as ‘the minimal essential MHC’ [29]. This compact MHC structure has been reported in various bird lineages (e.g., [30-35]), but is not universal in birds. The Japanese quail (*Coturnix japonica*) appears to have a more complex MHC structure [36] than the chicken, even though the two belong to the same family. A highly complex MHC structure has been reported in passerine species, which commonly have many copies of both class I and II MHC genes and a large number of pseudogenes (e.g., [37-40]). The considerable variation among bird taxa suggests that the avian MHC structure is evolutionarily labile, probably due to recent gene duplications and pseudogene formation [41].

In the present study, we investigated the genetic diversity of the MHC class IIβ loci in Hokkaido fish owl populations, based on samples collected from 1963 to 2012. We used massively parallel pyrosequencing [42,43] for exhaustive genotyping of the fish owl MHC class IIβ loci. Our aims were (1) to describe the polymorphism at second exon of MHC class IIβ genes in the fish owl, and (2) to elucidate spatial and temporal patterns in the MHC diversity in order to assess how the recent population decline associated with habitat loss and fragmentation have affected variation in these functional genes.

## Materials and methods

### Sampling and DNA extraction

From 1963 to 2012, blood or skin tissue samples were collected from 200 individuals of the fish owl from five geographically isolated populations across Hokkaido: Shiretoko (SR), Konsen (KS), Akan (AK), Daisetsu (DS), and Hidaka (HD) populations (Figure 1). Most samples were from first-year juveniles, though some were from adults. All blood samples were non-invasively collected from owls by a veterinarian in the activity for conservation of the Blakiston’s fish owl by the Ministry of the Environment, Japan. Some drops of the blood samples were preserved in 99.9% ethanol or dried on filter paper, and stored at −20°C. Fibroblasts were cultured from skin tissue samples according to the method described in Nishida et al. [44]. Total genomic DNA was extracted from blood and fibroblasts with the DNeasy Blood & Tissue Kit (Qiagen).

### RNA extraction and cDNA synthesis

For expression analysis, total RNA was extracted with the RNeasy Mini Kit (Qiagen) from skin fibroblasts from 16 Hokkaido individuals. First-strand cDNA was synthesized from 1 μg of total RNA using the oligo-(dT)_20_ primer with the Superscript III First-strand Synthesis System (Invitrogen). The expression of MHC class II molecules in skin fibroblasts was confirmed through polymerase chain reaction (PCR) using the primers Sf-ex1F (5′-CAC TGG TGG TGC TGG GAG CC-3′) and Tyal-ex3-3′R (5′-AGG CTG ACG TGC TCC ACC TG-3′), which bind in conserved regions of exons 1 and 3 of owl MHC class II loci [32,45].

### Primer design

To develop specific primers for pyrosequencing, complete and partial sequences of the second exon of the MHC class IIβ loci were amplified from several individuals from different populations in Hokkaido and from different years by using the primer sets Sf-ex1F and Tyal-ex3-3′R, and Stri2FC (5′-CMC ACA CAG GGG TTT TCC-3′) and Stri2RC (5′-AAC GYG YGG CCA CGC GCT CA −3′) [30]. Six cDNA and 10 genomic DNA samples were used as PCR templates. PCR amplifications were performed in 25-μl reaction volumes, each containing 0.5 units of high-fidelity PrimeSTAR GXL DNA Polymerase (TaKaRa Bio), 1 × PrimeSTAR GXL buffer, 200 μM each dNTP, 1 μM each primer, and approximately 200 ng of cDNA or 50 ng of genomic DNA. Cycling conditions were 98°C/1 min, 28 × (98°C/10 s, 68°C/30 s), 68°C/3 min for Sf-ex1F and Tyal-ex3-3′R; and 98°C/1 min, 28 × (98°C/10 s, 58°C/10 s, 68°C/30 s), 68°C/3 min for Stri2FC and Stri2RC. Because the genomic sequences of the MHC class IIβ region are extraordinarily GC-rich in avian species, we used high annealing temperature to increase the efficiency of amplifications as recommended in Burri et al. [46]. PCR products were cloned using the Zero Blunt TOPO PCR Cloning Kit (Invitrogen), and 24–48 clones per sample were sequenced with the BigDye Terminator v3.1 Cycle Sequencing Kit (Applied Biosystems) and an ABI 3730 DNA Analyzer (Applied Biosystems). From consensus sequences for the sequences obtained, specific primers were designed to amplify part of the second exon of the fish owl MHC class IIβ loci.

### Preparation of an amplicon library and pyrosequencing

Pyrosequencing was performed on 242 amplicons obtained from 200 genomic DNA samples and 16 cDNA samples. To generate an amplicon library, fusion primers were designed that contained the GS FLX Titanium primer sequence (A in forward, B in reverse primers) at the 5′ end, followed by a 10-bp multiplex identifier (MID) sequence and the sequence of a gene-specific primer. The MID sequences were chosen from the Extended MID Set (Roche) and were used to distinguish amplicons obtained from different PCR reactions. For genomic DNA samples, newly designed primers BublIIb2F (5′-GAG TGT CAG YAC CTY RAY RG-3′) and BublIIb2R (5′-CTT TCY TCT SCS TGA YGW AGG-3′) were used as forward and reverse gene-specific primers to amplify 203-bp fragments of the second exon of MHC class IIβ loci ([see Additional file 1: Figure S1]). Ten genomic DNA samples were amplified and sequenced twice to estimate the genotyping error. For cDNA samples, we used 2 reverse primers (BublIIb2-3R1, 5′-TTC CAC CTC GGG CGG GAC TTT C-3′; BublIIb2-3R2, 5′-CCT CAC CTT GGG CTG AAC TTT C-3′) that spanned the exon-exon junction between the second and third exons. PCR was conducted twice for each cDNA sample to amplify 213-bp fragments, using the fusion primer pairs containing the sequences of BublIIb2F/BublIIb2-3R1 and BublIIb2F/BublIIb2-3R2.

PCR conditions were 98°C/1 min, 28 × (98°C/10 s, 58°C/10 s, 68°C/30 s), and 68°C/3 min. PCR amplicons were purified by using the MinElute PCR Purification Kit (Qiagen). After quantification by agarose-gel electrophoresis, the purified amplicons were pooled in approximately equimolar quantities. The amplicon library was commercially sequenced on a 1/4 Titanium Pico Tire Plate with the GS FLX Titanium Sequencing Kit XLR70 (Roche) at Hokkaido System Science Co. (Sapporo, Japan).

### MHC genotyping

SFF Tools 2.8 (Roche) was used to assign the processed reads to respective individuals based on the MID sequences in the forward and reverse fusion primers, and seq_crumbs 0.1.8 (available from http://bioinf.comav.upv.es/seq_crumbs/) was used to trim and filter sequence reads based on quality and sequence length.

Allele detection and genotyping were performed with the recently developed pipeline ngs_genotyping 0.9.0 ([47]; available from https://github.com/enormandeau/ngs_genotyping). This pipeline uses an iterative procedure in three successive steps. The first step generates putative allele sequences for each individual. The second step combines and strengthens these into the global alleles for all individuals. The third step then genotypes each individual. Prior to these steps, sequence reads were iteratively cleaned and aligned by means of the MUSCLE algorithm [48]. Throughout the text, unique sequence variants are referred to as ‘alleles’ for convenience, although this is not strictly correct, as these sequences represent multiple loci.

The following parameters were used with a hierarchical clustering analysis in the first step of the pipeline to filter out sequencing errors and to detect putative alleles: minimum internal branch length, 0.06; minimal proportion threshold to define the cluster, 0.02. Likewise, the following parameters were used to detect global consensus alleles in the second step: minimum internal branch length, 0.06; minimum number to define the cluster, two sequences. In the third step, the number of sequence reads of each global allele was counted for each individual by using the BLASTn algorithm. Each individual was then genotyped according to the minimal proportion threshold of each allele. Threshold values ranging from 0.01 to 0.05 were used, depending on natural breaks in the number of reads per individual for each allele. The above settings of the threshold values in the pipeline meet the two-PCR criterion that is standard in MHC studies [49].

### Data analysis

Phylogenetic relationships among the fish owl MHC class IIβ alleles were reconstructed by Bayesian inference and maximum likelihood (ML) analyses, using the programs MrBayes 3.2.2 [50] and GARLI 2.01 [51]. The best-fit model of nucleotide substitution was selected based on the Bayesian information criterion implemented in jModelTest 2.1.1 [52]. MrBayes analyses were performed with two parallel runs of 20 million generations each and using one cold and three heated Markov chains, with sampling every 1000 steps. Convergence and stationarity of the chains were confirmed by the average standard deviation of split frequencies (<0.01). The first 25% of trees were discarded as burn-in, and a 50% majority-rule consensus tree was constructed from the remaining trees. An ML bootstrap search with 1000 pseudo-replicates were perfomed with GARLI. For these analyses, sequences of the MHC class IIβ alleles of northern goshawk (*Accipiter gentilis*; EF370917) and Eurasian black vulture (*Aegypius manachus*; EF370890) were also included as outgroups. An additional analysis was conducted to reconstruct the relationships between MHC class IIβ alleles identified in the fish owl and those in other owl species (GenBank accession nos. EF370927–370928, EF370930–370946, and EF641223–EF641262 [30,32]).

A *Z*-test for selection was performed to detect the signature of historical selection on the fish owl MHC class IIβ loci by using MEGA 5.2.2 [53] with the modified Nei-Gojobori method and Jukes-Canter correction. The average rates of synonymous (*d*_S_) and non-synonymous substitution (*d*_N_) were calculated for all sites, as well as for positions encoding amino acids in the putative peptide-binding region (PBR), and the remaining positions (putative non-PBR). The location of the PBR was inferred from the molecular structure of human leukocyte antigen class II, *HLA-DR1* [54]. In addition, Bayesian inference was performed with omegaMap 0.5 [55] to detect amino acid sites under positive selection. This program uses a coalescent-based model for detecting natural selection in the presence of recombination, and uses the reversible-jump Markov chain Monte Carlo to perform Bayesian inference of both the *d*_N_*/d*_S_ ratio (ω) and the recombination rate (ρ). The omega_model was set to independent, with the rho_model set to variable and the rho_block set to 3. To reduce computation time, two independent random subsamples of 200 alleles each were generated from all populations. Three independent runs of 2 million steps each were performed for each subsample. Convergence and stationarity of the runs were assessed by calculating the effective sample size, which exceeded 200 for all estimated parameters. The first 50,000 steps were discarded as burn-in. Codons were considered to be under positive selection if the posterior probability of ω > 1 exceeded 0.95 in both independent subsample run sets.

The detection of potential recombinant sequences in our data set was carried out using a set of seven non-parametric detection methods implemented in RDP4 beta 4.27 software [56]: RDP, GENECONV, MaxChi, Chimaera, BootScan, SiScan, and 3Seq. The analysis was performed with default settings for the various detection methods, and the Bonferroni-corrected *P* value cutoff was set at 0.05. Recombination events were accepted when detected with at least three of the seven detection methods. Additionally, the web-based service GARD (genetic algorithm for recombination detection) [57] was used to detect recombination breakpoints.

Population-level allelic richness was calculated through 1000 bootstrap replicates with a constant sample size (*n* = 4). Nucleotide diversity (*π*) within individuals and populations were calculated using MEGA. To assess the MHC diversity within and between populations, R 3.0.1 [58] with the package ecodist 1.2.7 [59] was used to calculate Jaccard distances from a binary presence/absence matrix for each allele in pairwise comparisons of individuals. Jaccard dintance is the measure of dissimilarity between sample sets, and is defied as one minus ratio of the size of the intersection and union of the sample sets [60]. Non-metric multidimensional scaling (NMDS) was performed to visualize the relationships among individual MHC genotypes based on Jaccard distances.

To assess the genetic differentiation among populations, global and population pairwise *G*_ST_ values [61] were estimated from allele frequencies, using the mmod package version 1.2.1 [62] in R. The significance of the *G*_ST_ values were evaluated with the permutation tests (1000 replicates). Finally, an analysis of molecular variance (AMOVA) [63] was conducted to evaluate the spatial and temporal patterns of genetic variations, using the ade4 package version 1.6-2 [64] in R. An AMOVA was performed with two different genetic distance matrixes calculated in pairwise comparison of individuals: 1) Jaccard distance, and 2) average number of nucleotide substitutions per site between individuals, calculated with the Kimura 2-parameter model. The total genetic variance was partitioned into three hierarchical levels: among populations, between periods within populations, and within periods. The significance of the variance components were assessed with the permutation tests (1000 replicates).

## Results

### Genotyping of fish owl MHC class IΙβ

Amplification primers with the complete MID sequences were identified in 193,393 reads. Final genotypes were based on 139,832 reads; the mean coverage (number of reads per amplicon) was 577.8 reads (SD = 307.7, range = 34–1851). For conservative genotyping, individuals with fewer than 200 reads were excluded from the analysis. No correlation between the number of reads and the number of alleles observed per individual was detected in the remaining samples ([see Additional file 1: Figure S2]), indicating that the coverage was sufficient for reliable genotyping. Of the 10 replicated pairs that were run twice to estimate genotyping error, nine pairs had > 200 reads for both replicates, and the same alleles were found in these pairs. In all, 174 individuals were genotyped (Table 1; Additional file 2).

**Table 1:** **Allelic richness and nucleotide diviersity at MHC class IIβ genes in the fish owl populations**

**Period**	**Population**	**N**	***K***_**individ**_	***K***_**pop**_	***AR***	***π***_**individ**_	***π***_**pop**_
1963–1992	SR	11	12	16	14.99	0.159 ± 0.014	0.147 ± 0.013
	KS	15	11	14	11.71	0.161 ± 0.015	0.148 ± 0.013
	AK	7	11	16	13.63	0.161 ± 0.016	0.149 ± 0.014
	DS	4	11	16	14.42	0.162 ± 0.015	0.152 ± 0.014
1993–2002	SR	29	12	18	14.96	0.158 ± 0.014	0.146 ± 0.013
	KS	11	11	11	11.00	0.160 ± 0.014	0.147 ± 0.013
	AK	6	11	12	11.79	0.161 ± 0.015	0.149 ± 0.013
	DS	11	12	15	13.77	0.161 ± 0.014	0.150 ± 0.013
2003–2012	SR	29	12	19	15.93	0.158 ± 0.014	0.146 ± 0.013
	KS	12	11	11	11.00	0.161 ± 0.015	0.148 ± 0.013
	AK	9	10	12	11.18	0.165 ± 0.015	0.151 ± 0.014
	DS	18	12	16	14.28	0.161 ± 0.014	0.149 ± 0.014
	HD	12	13	17	15.90	0.159 ± 0.015	0.149 ± 0.014

N, sample size; *K*_individ_, median number of different alleles per individual; *K*_pop_, number of different alleles in the sampled population; *AR*, population-level allelic richness calculated via bootstrapping with a constant sampling size (N = 4); *π*_individ_, mean nucreotide diversity (± SE) within individuals; *π*_pop_, mean nucleotide diversity (± SE) within poplations. Population abbreviations: SR, Shiretoko; KS, Konsen; AK, Akan; DS, Daisetsu; HD, Hidaka.

### Phylogenetic relationships among MHC alleles and allelic expression patterns

From the partial sequences (203 bp) of MHC class IIβ exon 2 among 174 fish owls, 19 unique variants (alleles) were identified. We named these alleles Bubl-DAB*01–19 (only the abbreviation Bubl is used hereafter), following the nomenclature proposed by Klein et al*.* [65]. Sequences of all alleles were deposited in the DNA Data Bank of Japan (DDBJ) under accession nos. LC007937–LC007955 (sequence alignment in Additional file 3).

**Figure 2:**
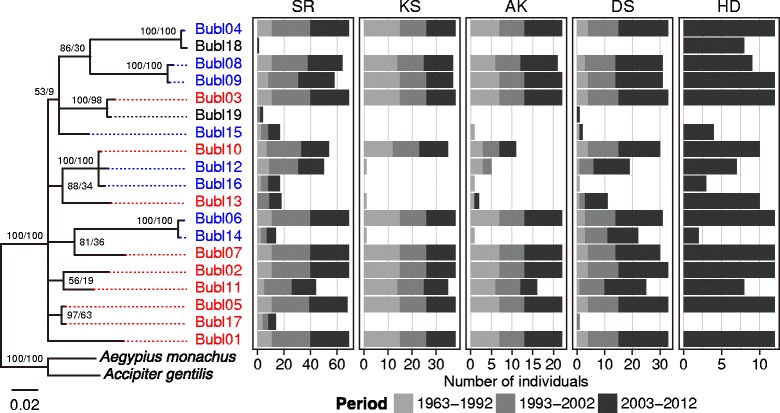
**Phylogenetic relationship and spatio-temporal distribution of fish owl MHC class IIβ allleles.** Fifty percent majority-rule Bayesian consensus tree of fish owl MHC class IIβ alleles Bubl01–19 (leftmost of the figure) were constructed under Kimura 2-parameter model with gamma-distributed rate variation among sites using MrBayes. Sequences of the MHC class IIβ alleles of northern goshawk (*Accipiter gentilis*; EF370917) and Eurasian black vulture (*Aegypius manachus*; EF370890) were included as outgroups. Numbers at nodes are Bayesian popstrior porobabilities (left) and ML bootstrap support values (right) for MrBayes and GARLI analyses. Alleles obtained from cDNA samples are shown in red, and that obtained from genomic DNA but not in cDNA are shown in blue. Alleles whose expression status are unknown are shown in black. The scale bar indicates branch length in substitutions per site. Bar charts on the right side of the tree show spatial and temporal distribution of the alleles. Population abbreviations: SR, Shiretoko; KS, Konsen; AK, Akan; DS, Daisetsu; HD, Hidaka.

The alleles we detected showed high degrees of nucleotide (94/203 sites variable) and amino acid (43/67 sites variable) polymorphism. The number of alleles per individual ranged from eight to 16, indicating there are at least eight MHC class IIβ loci in this species. Nine of 19 alleles were also found in 16 cDNA samples (Figure 2 and [see Additional file 1: Table S1]). Up to eight alleles were detected in cDNA samples from single individuals, indicating that at least four loci are expressed in skin fibroblasts.

Additional file 1: Figure S3 shows the phylogenetic relationships among MHC class IIβ alleles from the fish owl and other owls. This pattern is one of trans-species polymorphism [66]: alleles in one species are most closely related to alleles in other species, rather than grouping by species. No alleles were shared between the fish owl and other owl species based on the nucleotide sequences or amino acid sequeneces.

### Patterns of selection and recombination

Because the allele Bubl12 contains an internal stop codon ([see Additional file 1: Figure S4]), this allele was excluded from the *Z*-test and the omegaMap analysis. A highly significant excess of non-synonymous over synonymous substitutions was observed for codons in the putative peptide-binding regions (PBR), whereas synonymous substitutions were more frequent than non-synonymous substitutions in non-PBR codons (Table 2). The similar patterns were obtained in the analyses perfomed only with expressed alleles and that only with unexpressed alleles, although more synonymous and non-synonymous mutations were detected in the comparisons between the unexpressed alleles than between the expressed alleles (Table 2). The omegaMap analysis indicated that 13 of 67 amino acid positions are under balancing selection ([see Additional file 1: Figure S5]). These positions lined up with 10 of 19 putative PBR residues defined by alignment to human *HLA-DR1* ([see Additional file 1: Figure S4]). In addition, 2 of the putative non-PBR residues had a signature of balancing selection. No evidence of recombination were found within exon 2 sequences of the fish owl MHC class IIB alleles in either RDP or GARD analyses.

**Table 2:** **Results of** ***Z*** **-tests for selection on fish owl MHC class IIβ sequences**

	***d***_**S**_	***d***_**N**_	***Z***	***P***
(A) All alleles				
PBR	0.139 ± 0.059	0.464 ± 0.091	3.563	<0.001
Non-PBR	0.167 ± 0.040	0.095 ± 0.020	−1.644	1.000
All sites	0.158 ± 0.031	0.179 ± 0.028	0.309	0.499
(B) Expressed alleles				
PBR	0.109 ± 0.049	0.404 ± 0.081	3.100	0.001
Non-PBR	0.114 ± 0.033	0.058 ± 0.016	−1.579	1.000
All sites	0.112 ± 0.026	0.139 ± 0.026	0.801	0.212
(C) Unexpressed alleles				
PBR	0.161 ± 0.084	0.468 ± 0.105	2.589	0.005
Non-PBR	0.236 ± 0.074	0.127 ± 0.029	−1.413	1.000
All sites	0.213 ± 0.052	0.206 ± 0.034	−0.124	1.000

*Z*-test for selection was conducted with (A) all alleles together, (B) only with expressed alleles, and (C) only with unexpressed alleles (see Figure 2 and [Additional file 1: Table S1]). The average rates of synonymous (*d*_S_) and non-synonymous substitution (*d*_N_) were calculated for all sites, as well as for positions encoding amino acids in the putative peptide-binding region (PBR), and the remaining positions (putative non-PBR). Standard errors obtained through 1000 bootstrap replicates. The location of the PBR was inferred from the molecular structure of human leukocyte antigen class II, *HLA-DR1* [54].

### Spatial and temporal patterns of MHC diversity

**Figure 3:**
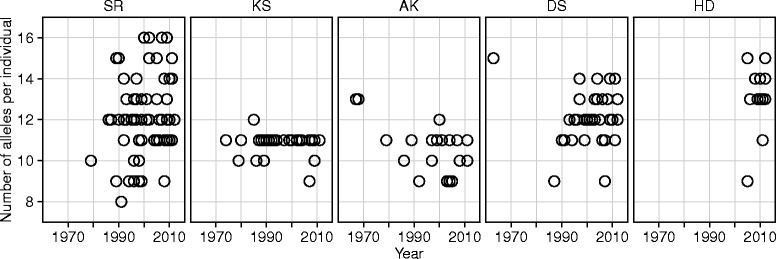
**Number of the MHC class IIβ alleles detected per individual, by population and year.** Each circle indicates one individual. Population abbreviations: SR, Shiretoko; KS, Konsen; AK, Akan; DS, Daisetsu; HD, Hidaka.

The variablity in the number of alleles per individual differed among populations (Figure 3), although there is little difference in the median number of alleles per indiviual among populations (Table 1). The SR population shows highest allelic richness in the all periods (Table 1), and all of the 19 alleles were observed in this population during the period of 2003–2012. A relatively high alleric richness was detected in HD in the period of 2003–2012 (Table 1). Although DS shows moderate allelic richness (Table 1), alleles Bubl16 and Bubl17, both of which were found before 2002, were not found thereafter. Conversely, Bubl19 was found only in individuals sampled after 2003 (Figure 2). The estimates of alleleic richness were decreased in KS and AK in the last several decades, and only 11 or 12 alleles were observed in these populations in the period of 2002–2012 (Table 1). Some alleles whose frequencies were initially low were probably lost in these populations (Figure 2). Meanwile, nucleotide diversities were relateively higher in the KS and AK populations, indicating that remained alleles in these populations were highly diverged from each other. Interestingly, these alleles were also maintained in high frequencies in the other populations (Figure 2).

**Figure 4:**
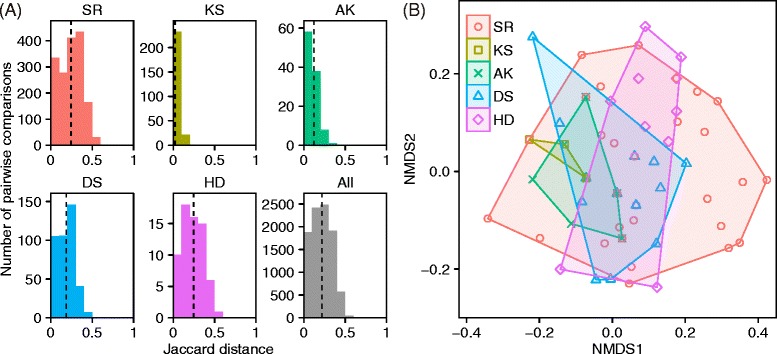
**Spatial patterns of MHC diversity in current populations of Blakiston’s fish owl. (A)** Frequency distribution of the Jaccard distance between individuals, calculated for each population using the samples collected from 1993 to 2012. Dashed lines indicate mean values. **(B)** Non-metric multidimensional scaling (NMDS) plot based on Jaccard distances, showing the relationships of individual MHC genotypes among populations. Population abbreviations: SR, Shiretoko; KS, Konsen; AK, Akan; DS, Daisetsu; HD, Hidaka.

Figure 4A shows the distribution of Jaccard distances among individuals within populations, calculated from samples collected from 1993 to 2012. Mean Jaccard values were 0.244 (SR), 0.023 (KS), 0.123 (AK), 0.188 (DS), 0.245 (HD), and 0.220 (All). The peaks of the distributions of Jaccard distance were near zero for KS and AK (Figure 4A), indicating that most individuals in these populations had a similar set of alleles. An NMDS plot shows that genotypic diversity was lower in these populations than in the other populations (Figure 4B). No significant genetic differences were detected among populations in the period of 1963–1992 (global *G*_ST_ = −0.0014, *P* = 0.949; median of pairwise *G*_ST_ = −0.0012 [see Additional file 1: Table S2]) or in the period of 1993–2002 (global *G*_ST_ = −0.0010, *P* = 0.927; median of pairwise *G*_ST_ = −0.0002), whereas slight but significant genetic differences were detected among poplations in the period of 2003–2012 (global *G*_ST_ = 0.0012, *P* = 0.039; median of pairwise *G*_ST_ = 0.0022). Hierachical analysis by AMOVA revealed that substantial amount of genetic variation was attiributed to differences among populations (Table 3). An AMOVA also detected weak but partially significant genetic differences among periods whitin populations (Table 3). Interestingly, AMOVA revealed a high proportion of variance explained between populations, while *G*_ST_ values were very small. This may be because *G*_ST_ only considers allele frequencies, and maximal *G*_ST_ is reduced for highly variable markers such as typically microsatellites, but also MHC [67].

**Table 3:** **Results of analysis of molecular variance (AMOVA)**

		**Jaccard distance**			**Kimura 2-parameter distance**		
**Source of variation**	**Df**	**Variance (%)**	**Φ statistics**	***P***	**Variance (%)**	**Φ statistics**	***P***
Between populations	4	0.024 (20.70)	0.207	<0.001	0.014 (17.57)	0.176	<0.001
Between periods within populations	8	0.002 (1.32)	0.017	0.050	0.001 (1.18)	0.014	0.093
Within periods	161	0.091 (77.98)	0.220	<0.001	0.067 (81.25)	0.188	<0.001

AMOVA was performed with two different genetic distance matrixes calculated in pairwise comparison of individuals: 1) Jaccard distance, and 2) average number of nucleotide substitutaions per site between individuals, calculated with the Kimura 2-parameter model.

## Discussion

### MHC class IIβ genes in Blakiston’s fish owl

Our results show that the Blakiston’s fish owl genome contains at least eight MHC class IIβ loci, among which at least four are expressed in skin fibroblasts. Although the number of individuals used in the expression analysis was insufficient to confirm the expression status of all alleles, some alleles isolated from genomic DNA samples were not detected in the cDNA samples from the same individuals (Figure 2, [see Additional file 1: Table S1]), suggesting that these alleles are pseudogenes or have different functions. Indeed, one of these alleles is clearly non-functional, as it contains an internal stop codon. The other alleles not detected in the cDNA samples are possibly expressed at very low levels or in tissues other than skin fibroblasts. Further study is required to clarify the actual number of gene copies and expression status of the fish owl MHC class IIβ genes.

The evolution of the genomic structure of avian MHC is of particular interest, as it varies considerably among taxa [41]. To our knowledge, the number of MHC class IIβ genes in the fish owl suggested by our results is the greatest in any non-passerine bird. Besides the fish owl, Burri et al. [45] reported that barn owl (*Tyto alba*) has two duplicated MHC class IIβ genes, by means of a Southern blot analysis. Although the number of MHC class IIβ genes has not yet been investigated in detail in other owls, the cDNA based study revealed that the strigiform owls have two or more gene copies [32,68]. Our results suggest significant variation in the number of gene copies in owl MHC class IIβ. It is unknown why the fish owl has a large number of MHC class IIβ genes. Although the reasons for the taxonomic trends in avian MHC structure are yet unclear, gene duplications may have occurred at a higher frequency in some lineages than others due to the activity of endogenous retroviral elements, as has been suggested for primate [69], passerine [38], and wallaby MHC genes [70]. Future comparative genomic studies of the fish owl MHC structure using more powerful approaches, such as whole-genome sequencing or screening BAC libraries, will provide new insights into the evolutionary history of the avian MHC. From an eco-immunological point of view, the complexity of MHC genes may be related to the complexity of pathogens in the environment. As the fish owl specializes on aquatic prey, it may be exposed to different suites of pathogens than most other owls, which feed on terrestrial prey. However, little is known about pathogens infecting the fish owl, and further study is necessary.

The *Z*-test and the omegaMap analysis revealed that the ratio of non-synonymous to synonymous substitutions was significantly higher than expected in PBRs, based on the neutral model, but not in non-PBRs (Table 2; [see Additional file 1: Figure S5]), a pattern consistent with balancing selection [71,72]. The pattern of trans-species polymorphism further supports the conclusion that balancing selection acted in the past ([see Additional file 1: Figure S3]). These results are concordant with most other studies reporting selective signatures for MHC genes (reviewed in [14]).

### Effect of population reduction and fragmentation on fish owl MHC diversity

Although balancing selection might maintain MHC polymorphisms over the long term, strong genetic drift in populations that undergo a bottleneck and fragmentation can counteract the effects of balancing selection [25]. Our results indicate that a recent habitat loss and fragmentation has substantially lowered MHC diversity in the fish owl. Using massively parallel pyrosequencing, we identified 19 alleles among 174 individuals from Hokkaido populations. Assuming that there are at least eight loci, the level of genetic variation in MHC class IIβ loci is low in the fish owl populations in Hokkaido, as other species often show higher levels of MHC class IIβ allelic diversity. For example, Alcaide and colleagues [73] detected 103 alleles at a single locus among 121 individuals of the lesser kestrel (*Falco naumanni*). High levels of allelic diversity at MHC genes in wild bird populations were also reported in the recent studies using next-generation sequencing approach [39,40,74]. The low variation detected in the fish owl populations was not a consequence of limited sampling, as our population sample covered most families currently living in Hokkaido.

Our results also revealed that the MHC diversity differed among geographically isolated populations in Hokkaido. The highest allelic diversity was observed in the SR population (Table 1), in which all 19 alleles identified from all Hokkaido populations combined were observed during the last decade (Table 1 and Figure 2). NMDS analysis also detected high inter-individulal MHC diversity within SR populaion (Figure 4). These results correlate with the large size of the SR population; approximately half the fish owls in Hokkaido inhabit the Shiretoko Peninsula [3]. A microsatellite analysis also detected relatively high genetic diversity in this population [8]. A moderate level of MHC diversity was observed in the DS population, which is the second largest Hokkaido. MHC diversity was also relatively high in the HD population, despite its small size. Interestingly, this is not congruent with microsatellite data [8,9], where the HD population showed the lowest genetic diversity. This discrepancy suggests that analyses of neutral genetic markers alone inadequately quantify genetic variation. In contrast, inter-individulal MHC diversity was very low in the AK population and nearly lacking in the KS population (Figure 4). In the past two decades the number of alleles per individual in these populations was as high as 11 or 12 (Figure 3), which corresponded to the total number of alleles observed in these populations (Table 1). Assuming that there are at least eight loci, this result indicates that more than half the MHC class IIβ loci are homozygous in individuals in the AK and KS populations. Both populations are relatively small in size, and during the 1980s there may have been as few as one to several breeding pairs in each population. As we detected more alleles in individuals in these populations sampled before 1992 (Table 1 and Figure 2), the currently low MHC variation appears to be due to genetic drift associated with recent habitat loss and fragmentation.

Finaly, we did not investigate samples from a continental population in the present study. As the fish owl habitat is better preserved and population sizes are larger on the continent than in Hokkaido, higher MHC diversity may have been maintained in these population. Further analysis on a continental population may lead to a better understanding of the extent of MHC diversity in this species.

## Conclusions

Our study demonstrated substantial spatial variation in MHC diversity in the current fish owl populations on Hokkaido. Analysis of mtDNA sequences from historical specimens suggested that the genetic diversity was higher before the 1980s, and that before the middle of 20th century, there was gene flow over broad areas of Hokkaido [9]. The current spatial pattern in MHC diversity may thus have been shaped by loss of variation due to genetic drift in the fragmented populations. Low MHC variation among individuals, as in the KS and AK populations, should make these populations more vulnerable to epidemics of novel pathogens. The effects of low MHC diversity on the viability of fish owl populations remains unclear, however, as information on the abundance of pathogens and prevalence of diseases in wild fish-owl populations is scarce. Based on the results of recent population genetic studies, genetic rescue of the fish owl populations by translocation of outbred individuals has been proposed, and the results of our study will be useful in establishing such translocation plans. Although translocations could increase the adaptive genetic variation, a detailed screening of pathogens should be done before any attempts at mixing different populations are considered.

### Availability of supporting data

Sequences of the MHC class IIβ alleles are available in GenBank/EMBL/DDBJ accession nos. LC007937–LC007955.

Sequence alignment and genotyping data are included in the additional files.

## Additional files

Additional file 1: Tables S1, S2
**and** **Figures S1–S5.**
Additional file 2:
**MHC class IΙβ genotyping data of Blakistion’s fish owl populations.**
Additional file 3:
**Sequence alignment of Blakistion’s fish owl MHC class IIβ alleles (FASTA format).**

## References

[1] BirdLife International 2013. *Bubo blakistoni*. The IUCN red list of threatened species. version 2014.2. [http://www.iucnredlist.org]

[2] Slaght JC, Surmach SG (2008). Biology and conservation of Blakiston’s fish owls (*Ketupa balakistoni*) in Russia: a review of the primary literature and assessment of the secondary literature. J Raptor Res.

[3] Takenaka T (1998). Distribution, habitat environments, and reasons for reduction of the endangered Blakiston’s fish owl in Hokkaido, Japan. PhD thesis.

[4] Slaght JC, Surmach SG, Gutiérrez RJ (2013). Riparian old-growth forests provide critical nesting and foraging habitat for Blakiston’s fish owl Bubo blakistoni in Russia. Oryx.

[5] Takenaka T, Shiretoko Museum (1999). Shimafukuro. Birds in Shiretoko.

[6] Hayashi Y (1999). Past and present distribution of Blakiston’s fish owl (*Ketupa blakistoni*) in Hokkaido, Japan –based upon museum specimens–. J Yamashina Inst Ornithol.

[7] Brazil MA, Yamamoto S, Meyburg B-U, Chancellor RD (1989). The status and distribution of owls in in Japan. Raptors in the Modern World.

[8] Omote K, Nishida C, Takenaka T, Masuda R (2012). Temporal changes of genetic population structure and diversity in the endangered Blakiston’s fish owl (*Bubo blakistoni*) on Hokkaido Island, Japan, revealed by microsatellite analysis. Zoolog Sci.

[9] Omote K, Nishida C, Takenaka T, Saito K, Shimura R, Fujimoto S, Sato T, and Masuda R. Recent fragmentation of the endangered Blakiston’s fish owl (*Bubo blakistoni*) population on Hokkaido Island, northern Japan, revealed by mitochondrial DNA and microsatellite analyses. Zoolog Lett. in press. 10.1186/s40851-015-0014-3 PMC4657211 26605061

[10] Klein J (1986). Natural History of the Major Histocompatibility Complex.

[11] Garrigan D, Hedrick PW (2003). Perspective: detecting adaptive molecular polymorphism: lessons from the MHC. Evolution.

[12] Ohta T (1991). Role of diversifying selection and gene conversion in evolution of major histocompatibility complex loci. Proc Natl Acad Sci U S A.

[13] Nei M, Rooney AP (2005). Concerted and birth-and-death evolution of multigene families. Annu Rev Genet.

[14] Bernatchez L, Landry C (2003). MHC studies in nonmodel vertebrates: what have we learned about natural selection in 15 years?. J Evol Biol.

[15] Sommer S (2005). The importance of immune gene variability (MHC) in evolutionary ecology and conservation. Front Zool.

[16] Piertney SB, Oliver MK (2006). The evolutionary ecology of the major histocompatibility complex. Heredity (Edinb).

[17] Spurgin LG, Richardson DS (2010). How pathogens drive genetic diversity: MHC, mechanisms and misunderstandings. Proc R Soc B Biol Sci.

[18] Bollmer JL, Vargas FH, Parker PG (2007). Low MHC variation in the endangered Galápagos penguin (*Spheniscus mendiculus*). Immunogenetics.

[19] Zhu L, Ruan X-D, Ge Y-F, Wan Q-H, Fang S-G (2007). Low major histocompatibility complex class II DQA diversity in the Giant Panda (*Ailuropoda melanoleuca*). BMC Genet.

[20] Castro-Prieto A, Wachter B, Sommer S (2011). Cheetah paradigm revisited: MHC diversity in the world’s largest free-ranging population. Mol Biol Evol.

[21] Babik W, Kawalko A, Wójcik JM, Radwan J (2012). Low major histocompatibility complex class I (MHC I) variation in the European bison (*Bison bonasus*). J Hered.

[22] Biedrzycka A, Radwan J (2008). Population fragmentation and major histocompatibility complex variation in the spotted suslik, *Spermophilus suslicus*. Mol Ecol.

[23] Říčanová Š, Bryja J, Cosson J-F, Gedeon C, Choleva L, Ambros M (2011). Depleted genetic variation of the European ground squirrel in Central Europe in both microsatellites and the major histocompatibility complex gene: implications for conservation. Conserv Genet.

[24] Strand TM, Segelbacher G, Quintela M, Xiao L, Axelsson T, Höglund J (2012). Can balancing selection on MHC loci counteract genetic drift in small fragmented populations of black grouse?. Ecol Evol.

[25] Radwan J, Biedrzycka A, Babik W (2010). Does reduced MHC diversity decrease viability of vertebrate populations?. Biol Conserv.

[26] Belov K (2012). Contagious cancer: lessons from the devil and the dog. BioEssays.

[27] Hughes AL (1991). MHC polymorphism and the design of captive breeding programs. Conserv Biol.

[28] Ujvari B, Belov K (2011). Major histocompatibility complex (MHC) markers in conservation biology. Int J Mol Sci.

[29] Kaufman J, Milne S, Göbel TWF, Walker BA, Jacob JP, Auffray C (1999). The chicken B locus is a minimal essential major histocompatibility complex. Nature.

[30] Alcaide M, Edwards SV, Negro JJ (2007). Characterization, polymorphism, and evolution of MHC class II B genes in birds of prey. J Mol Evol.

[31] Ekblom R, Sæther SA, Jacobsson P, Fiske P, Sahlman T, Grahn M (2007). Spatial pattern of MHC class II variation in the great snipe (*Gallinago media*). Mol Ecol.

[32] Burri R, Hirzel HN, Salamin N, Roulin A, Fumagalli L (2008). Evolutionary patterns of MHC class II B in owls and their implications for the understanding of avian MHC evolution. Mol Biol Evol.

[33] Hughes CR, Miles S, Walbroehl JM (2008). Support for the minimal essential MHC hypothesis: a parrot with a single, highly polymorphic MHC class II B gene. Immunogenetics.

[34] Kikkawa EF, Tsuda TT, Sumiyama D, Naruse TK, Fukuda M, Kurita M (2009). Trans-species polymorphism of the Mhc class II DRB-like gene in banded penguins (genus *Spheniscus*). Immunogenetics.

[35] Wang Z, Zhou X, Lin Q, Fang W, Chen X (2013). Characterization, polymorphism and selection of major histocompatibility complex (MHC) DAB genes in vulnerable Chinese egret (*Egretta eulophotes*). PLOS ONE.

[36] Shiina T, Hosomichi K, Hanzawa K (2006). Comparative genomics of the poultry major histocompatibility complex. Anim Sci J.

[37] Anmarkrud JA, Johnsen A, Bachmann L, Lifjeld JT (2010). Ancestral polymorphism in exon 2 of bluethroat (*Luscinia svecica*) MHC class II B genes. J Evol Biol.

[38] Balakrishnan CN, Ekblom R, Völker M, Westerdahl H, Godinez R, Kotkiewicz H (2010). Gene duplication and fragmentation in the zebra finch major histocompatibility complex. BMC Biol.

[39] Zagalska-Neubauer M, Babik W, Stuglik M, Gustafsson L, Cichoń M, Radwan J (2010). 454 sequencing reveals extreme complexity of the class II Major Histocompatibility Complex in the collared flycatcher. BMC Evol Biol.

[40] Sepil I, Moghadam HK, Huchard E, Sheldon BC (2012). Characterization and 454 pyrosequencing of Major Histocompatibility Complex class I genes in the great tit reveal complexity in a passerine system. BMC Evol Biol.

[41] Hess CM, Edwards SV (2002). The evolution of the major histocompatibility complex in birds. Bioscience.

[42] Babik W, Taberlet P, Ejsmond MJ, Radwan J (2009). New generation sequencers as a tool for genotyping of highly polymorphic multilocus MHC system. Mol Ecol Resour.

[43] Galan M, Guivier E, Caraux G, Charbonnel N, Cosson J-F (2010). A 454 multiplex sequencing method for rapid and reliable genotyping of highly polymorphic genes in large-scale studies. BMC Genomics.

[44] Nishida C, Ishijima J, Ishishita S, Yamada K, Griffin DK, Yamazaki T (2013). Karyotype reorganization with conserved genomic compartmentalization in dot-shaped microchromosomes in the Japanese mountain hawk-eagle (*Nisaetus nipalensis orientalis*, Accipitridae). Cytogenet Genome Res.

[45] Burri R, Niculita-Hirzel H, Roulin A, Fumagalli L (2008). Isolation and characterization of major histocompatibility complex (MHC) class II B genes in the Barn owl (Aves: *Tyto alba*). Immunogenetics.

[46] Burri R, Promerová M, Goebel J, Fumagalli L (2014). PCR-based isolation of multigene families: lessons from the avian MHC class IIB. Mol Ecol Resour.

[47] Pavey SA, Sevellec M, Adam W, Normandeau E, Lamaze FC, Gagnaire P-A (2013). Nonparallelism in MHCIIβ diversity accompanies nonparallelism in pathogen infection of lake whitefish (*Coregonus clupeaformis*) species pairs as revealed by next-generation sequencing. Mol Ecol.

[48] Edgar RC (2004). MUSCLE: a multiple sequence alignment method with reduced time and space complexity. BMC Bioinformatics.

[49] Babik W (2010). Methods for MHC genotyping in non-model vertebrates. Mol Ecol Resour.

[50] Ronquist F, Teslenko M, van der Mark P, Ayres DL, Darling A, Höhna S (2012). MrBayes 3.2: efficient Bayesian phylogenetic inference and model choice across a large model space. Syst Biol.

[51] Zwickl DJ (2006). Genetic algorithm approaches for the phylogenetic analysis of large biological sequence datasets under the maximum likelihood criterion. Ph.D. thesis.

[52] Darriba D, Taboada GL, Doallo R, Posada D (2012). jModelTest 2: more models, new heuristics and parallel computing. Nat Methods.

[53] Tamura K, Peterson D, Peterson N, Stecher G, Nei M, Kumar S (2011). MEGA5: molecular evolutionary genetics analysis using maximum likelihood, evolutionary distance, and maximum parsimony methods. Mol Biol Evol.

[54] Brown JH, Jardetzky TS, Gorga JC, Stern LJ, Urban RG, Strominger JL (1993). Three-dimensional structure of the human class II histocompatibility antigen HLA-DR1. Nature.

[55] Wilson DJ, McVean G (2006). Estimating diversifying selection and functional constraint in the presence of recombination. Genetics.

[56] Martin DP, Lemey P, Lott M, Moulton V, Posada D, Lefeuvre P (2010). RDP3: a flexible and fast computer program for analyzing recombination. Bioinformatics.

[57] Pond SLK, Posada D, Gravenor MB, Woelk CH, Frost SDW (2006). GARD: a genetic algorithm for recombination detection. Bioinformatics.

[58] R Core Team (2012). R: A Language and Environment for Statistical Computing.

[59] Goslee SC, Urban DL (2007). The ecodist package for dissimilarity-based analysis of ecological data. J Stat Softw.

[60] Jaccard P (1908). Nouvelles recherches sur la distribution florale. Bull Soc Vaud Sci Nat.

[61] Nei M (1973). Analysis of gene diversity in subdivided populations. Proc Natl Acad Sci U S A.

[62] Winter DJ (2012). MMOD: an R library for the calculation of population differentiation statistics. Mol Ecol Resour.

[63] Excoffier L, Smouse PE, Quattro JM (1992). Analysis of molecular variance inferred from metric distances among DNA haplotypes: application to human mitochondrial DNA restriction data. Genetics.

[64] Dray S, Dufour AB, Chessel D (2007). The ade4 package-II: Two-table and K-table methods. R News.

[65] Klein J, Bontrop RE, Dawkins RL, Erlich HA, Gyllensten UB, Heise ER (1990). Nomenclature for the major histocompatibility complexes of different species: a proposal. Immunogenetics.

[66] Klein J (1987). Origin of major histocompatibility complex polymorphism: the trans-species hypothesis. Hum Immunol.

[67] Whitlock MC (2011). *G*’_ST_ and *D* do not replace *F*_ST_. Mol Ecol.

[68] Burri R, Salamin N, Studer RA, Roulin A, Fumagalli L (2010). Adaptive divergence of ancient gene duplicates in the avian MHC class II β. Mol Biol Evol.

[69] Dawkins R, Leelayuwat C, Gaudieri S, Tay G, Hui J, Cattley S (1999). Genomics of the major histocompatibility complex: haplotypes, duplication, retroviruses and disease. Immunol Rev.

[70] Siddle HV, Deakin JE, Coggill P, Whilming LG, Harrow J, Kaufman J (2011). The tammar wallaby major histocompatibility complex shows evidence of past genomic instability. BMC Genomics.

[71] Hughes AL, Nei M (1988). Pattern of nucleotide substitution at major histocompatibility complex class I loci reveals overdominant selection. Nature.

[72] Hughes AL, Yeager M (1998). Natural selection at major histocompatibility complex loci of vertebrates. Annu Rev Genet.

[73] Alcaide M, Edwards SV, Negro JJ, Serrano D, Tella JL (2008). Extensive polymorphism and geographical variation at a positively selected MHC class II B gene of the lesser kestrel (*Falco naumanni*). Mol Ecol.

[74] Alcaide M, Muñoz J, Martínez-de la Puente J, Soriguer R, Figuerola J (2014). Extraordinary MHC class II B diversity in a non-passerine, wild bird: the Eurasian Coot *Fulica atra* (Aves: Rallidae). Ecol Evol.

