# Supplementary material for: Spatial and temporal variation at major histocompatibility complex class IIB genes in the endangered Blakiston’s fish owl
Source: Zoological Lett. 2015 Mar 25;1:13. doi: 10.1186/s40851-015-0013-4 (PMC4657285; doi:10.1186/s40851-015-0013-4)
Supplement: Additional file 1: Tables S1, S2 — and Figures S1–S5. [file 40851_2015_13_MOESM1_ESM.pdf]

Additional file1

Table S1 MHC class IIB alleles detected in the fish owl samples used in the expression analysis. Alleles observed in both genomic DNA and cDNA are shown in red; those found only in genomic DNA are shown in black.

Sample ID	Year	Region	Observed alleles	Number of alleles	
				Expressed	All
ID010	1985	KS	**01**, **02**, 03, 04, **05**, 06, 07, 08, 09, **10**, **11**, 12	5	12
ID011	1986	AK	**01**, 02, 03, 04, **05**, 06, 07, 08, 09, **11**, 12	3	10
ID012	1986	SR	**01**, **02**, **03**, 04, **05**, 06, 07, 08, 09, **10**, 12, **17**	6	13
ID014	1987	DS	**01**, **02**, **03**, 04, **05**, **07**, 09, **10**, 14	6	9
ID019	1988	KS	**01**, **02**, 03, 04, **05**, 06, 07, 08, 09, **10**, **13**	5	11
ID021	1989	SR	**01**, **02**, **03**, 04, **05**, 06, 07, 08, 09, **10**, 12, 14, **15**, 16, **17**	6	15
ID025	1989	AK	**01**, **02**, 03, 04, **05**, 06, 07, 08, 09, **10**, **11**	5	11
ID028	1990	KS	**01**, **02**, **03**, 04, **05**, 06, 07, 08, 09, **10**, **11**	6	11
ID034	1992	SR	**01**, **02**, 03, 04, **05**, 06, 07, 08, 09, **10**, **11**, 12	5	12
ID038	1992	KS	**01**, **02**, 03, 04, **05**, 06, 07, 08, 09, **10**, **11**	5	11
ID043	1993	SR	**01**, **02**, **03**, 04, 06, **07**, 08, 09, 12, 14, **15**, 16, **17**	6	13
ID046	1994	DS	**01**, **02**, **03**, 04, **05**, 06, 07, 08, 09, **10**, 14	5	11
ID047	1994	DS	**01**, **02**, **03**, 04, **05**, 06, **07**, 08, 09, **10**, **11**, 12, **13**, 14	8	14
ID058	1995	SR	**01**, **02**, **03**, 04, **05**, 06, 07, 08, **10**	5	9
ID059	1996	SR	**01**, **02**, 03, 04, **05**, 06, 07, 08, 09, **10**, **11**, 12	5	12
ID060	1996	DS	**01**, **02**, **03**, 04, **05**, 06, 07, 08, 09, **10**, **11**, 14	6	12

Table S2 Pairwise G_{ST} estimates (below diagonal) between the populations in the three periods. *P*-values (above diagonal) were obtained by permutation tests (1000 replications). Significant G_{ST} values ($P < 0.05$) are highlighted.

		Period1 (1963–1992)				Period2 (1993-2002)				Period3 (2003-2012)				
		SR	KS	AK	DS	SR	KS	AK	DS	SR	KS	AK	DS	HD
Period1	SR		0.013	0.876	0.967	0.969	0.071	0.451	0.557	1.000	0.112	0.045	0.556	0.264
(1963–1992)	KS	0.0018		0.995	0.411	0.043	1.000	1.000	0.287	0.049	1.000	0.969	0.024	0.004
	AK	–0.0009	–0.0014		0.789	0.902	0.910	1.000	0.776	0.913	0.913	0.922	0.724	0.207
	DS	–0.0016	0.0001	–0.0014		0.898	0.305	0.537	1.000	0.983	0.408	0.257	0.987	0.621
Period2	SR	–0.0006	0.0013	–0.0007	–0.0011		0.007	0.442	0.487	1.000	0.019	0.004	0.889	0.218
(1963–2002)	KS	0.0022	–0.0017	–0.0010	0.0003	0.0015		1.000	0.112	0.005	1.000	0.696	0.008	0.001
	AK	–0.0001	–0.0024	–0.0030	–0.0005	–0.0002	–0.0024		0.583	0.376	1.000	0.986	0.306	0.050
	DS	–0.0001	0.0005	–0.0007	–0.0026	–0.0002	0.0006	–0.0006		0.481	0.140	0.016	1.000	0.061
Period3	SR	–0.0009	0.0017	–0.0007	–0.0015	–0.0005	0.0021	0.0002	0.0000		0.004	0.001	0.563	0.155
(2003–2012)	KS	0.0021	–0.0016	–0.0009	0.0000	0.0016	–0.0019	–0.0022	0.0007	0.0021		0.877	0.011	<0.001
	AK	0.0023	–0.0009	–0.0011	0.0008	0.0028	–0.0005	–0.0017	0.0023	0.0027	–0.0008		<0.001	<0.001
	DS	0.0000	0.0016	–0.0005	–0.0017	–0.0003	0.0020	0.0002	–0.0011	–0.0001	0.0022	0.0034		0.116
	HD	0.0012	0.0035	0.0010	–0.0005	0.0006	0.0039	0.0023	0.0013	0.0003	0.0040	0.0051	0.0006	

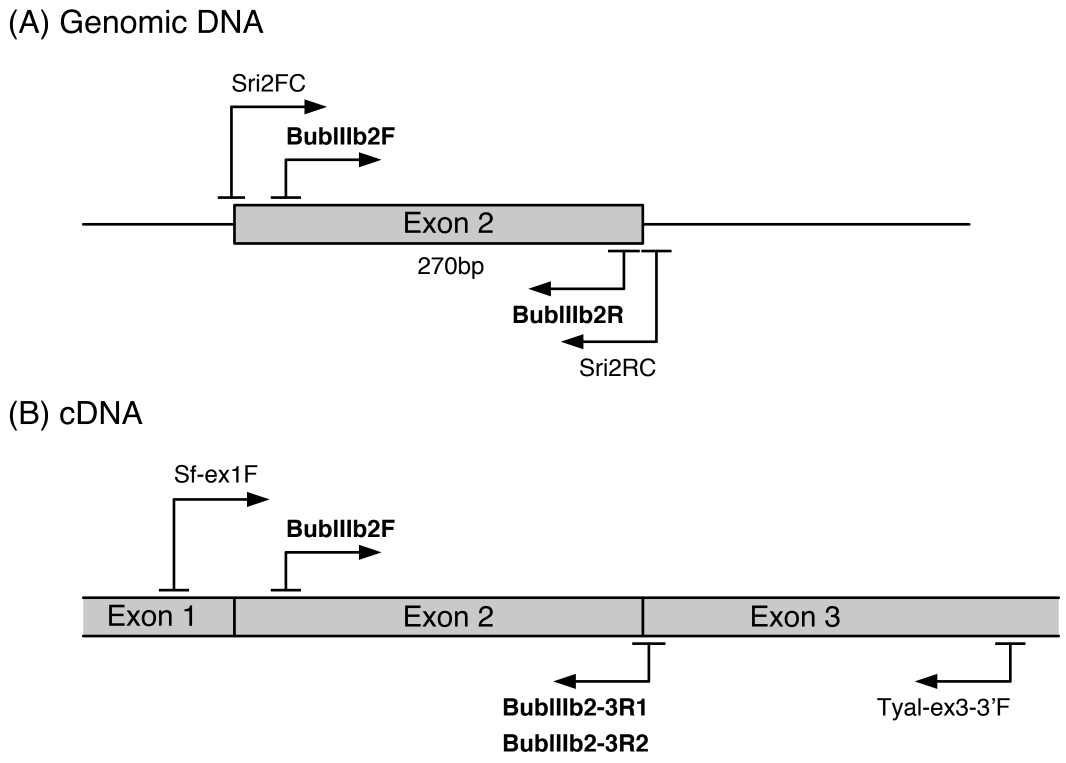

Figure S1 Location of primers used in the present study. Primer pairs used to amplify the fragments for pyrosequencing are shown in bold.

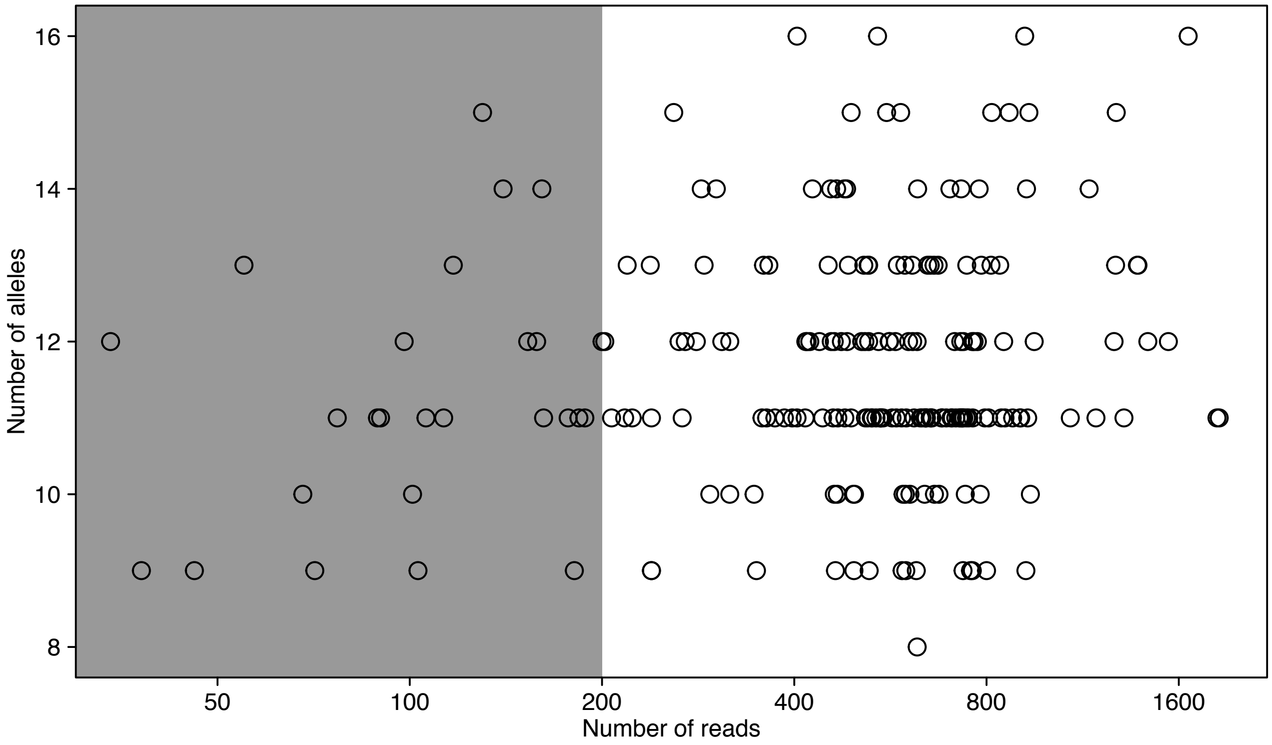

Figure S2 Relationship between the number of reads and the number of alleles observed per individual. Each circle indicates one individual. Individuals with fewer than 200 reads (background highlighted gray) were excluded from following analyses. No correlation between the number of reads and the number of alleles observed per individual was detected in the remaining samples (GLM with a Poisson-distributed error and log-link function; $\chi^2 = 0.863$; $P = 0.35$).

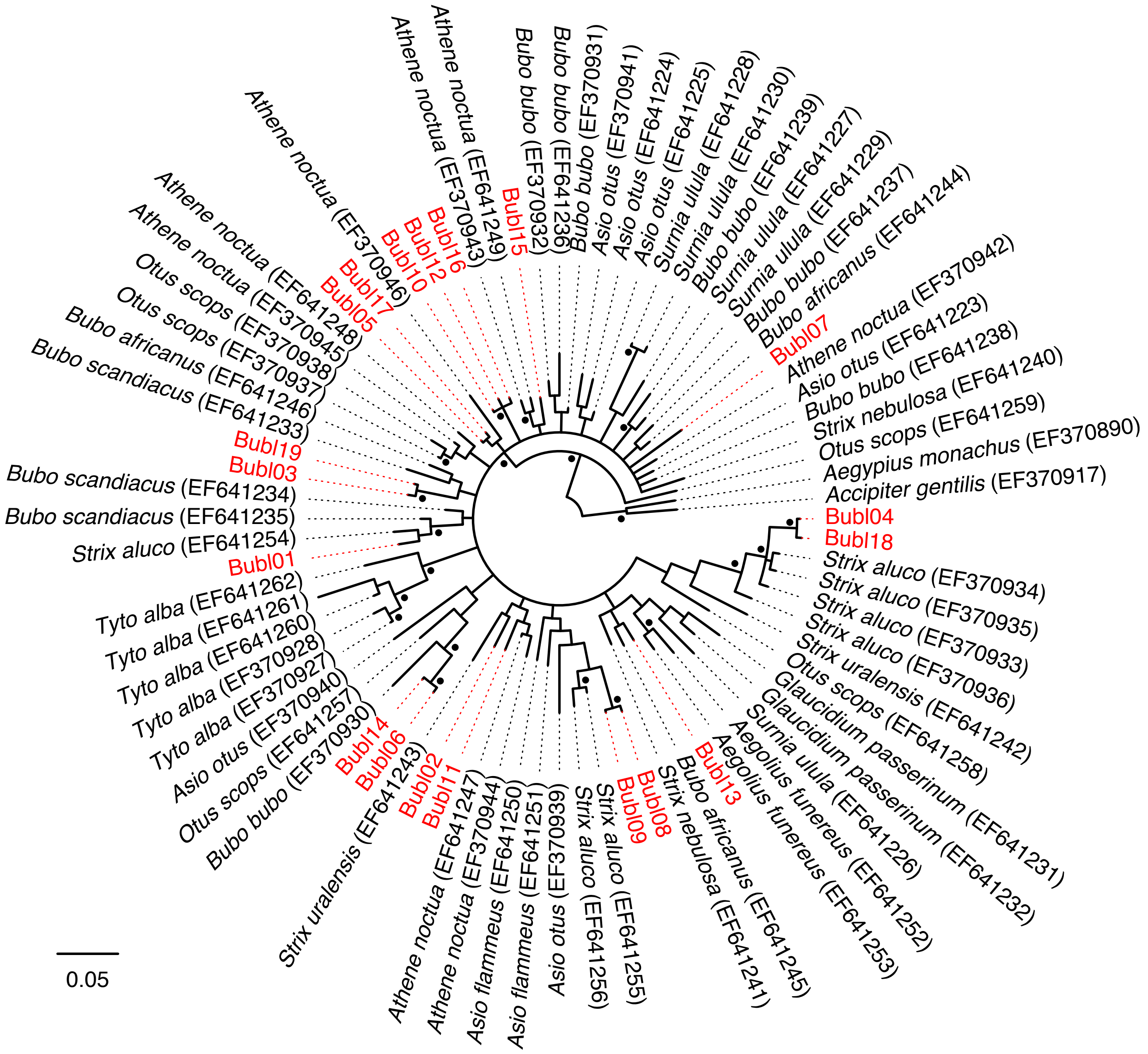

Figure S3 Fifty percent majority-rule Bayesian consensus tree showing relationships among MHC class IIβ alleles from the fish owl (in red) and from other owl species (GenBank accession nos. EF370927–370928, EF370930–370946, and EF641223–EF641262), constructed under Kimura's 2-parameter model with gamma-distributed rate variation among sites using MrBayes. Sequences of the MHC class IIβ alleles of northern goshawk (*Accipiter gentilis*; EF370917) and Eurasian black vulture (*Aegypius manachus*; EF370890) were included as outgroups. Filled circles indicate Bayesian posterior probabilities ≥ 90 as well as ML bootstrap values ≥ 70. The scale bar indicates branch length in substitutions per site.

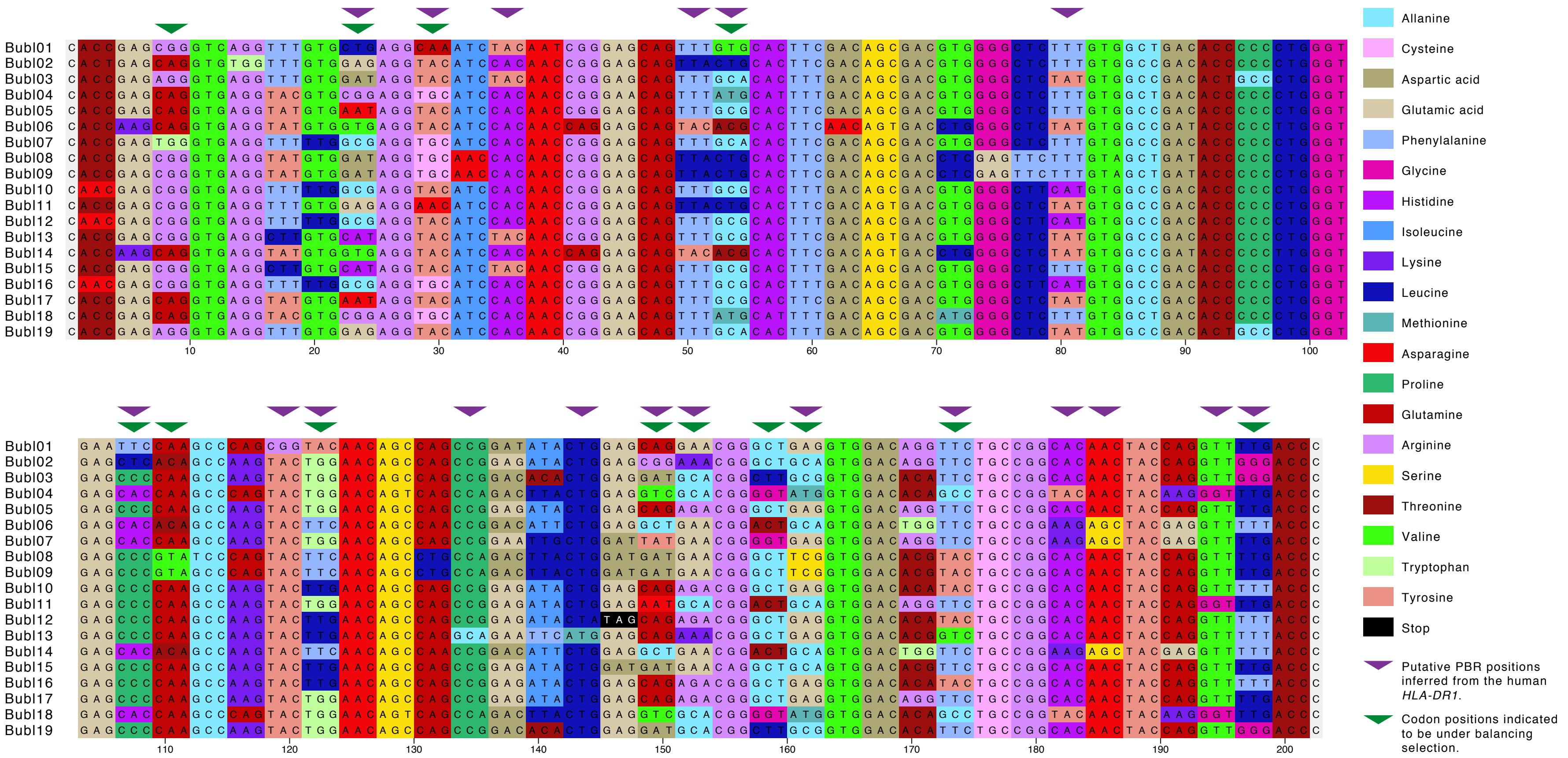

Figure S4 Alignment of nucleotide sequences of fish owl MHC class IIβ alleles. Codons are colored according to the amino acids they encode. Codon positions for the putative peptide binding region (PBR) inferred from the structure of the human *HLA-DR1* molecule [54] are indicated with purple arrows. Codon positions indicated by the omegaMap analysis to be under balancing selection from are indicated with green arrows. The figure was created with the Chromaseq package version 1.12 in Mesquite 3.01 (Available from http://mesquiteproject.org/packages/chromaseq/manual/index.html/).

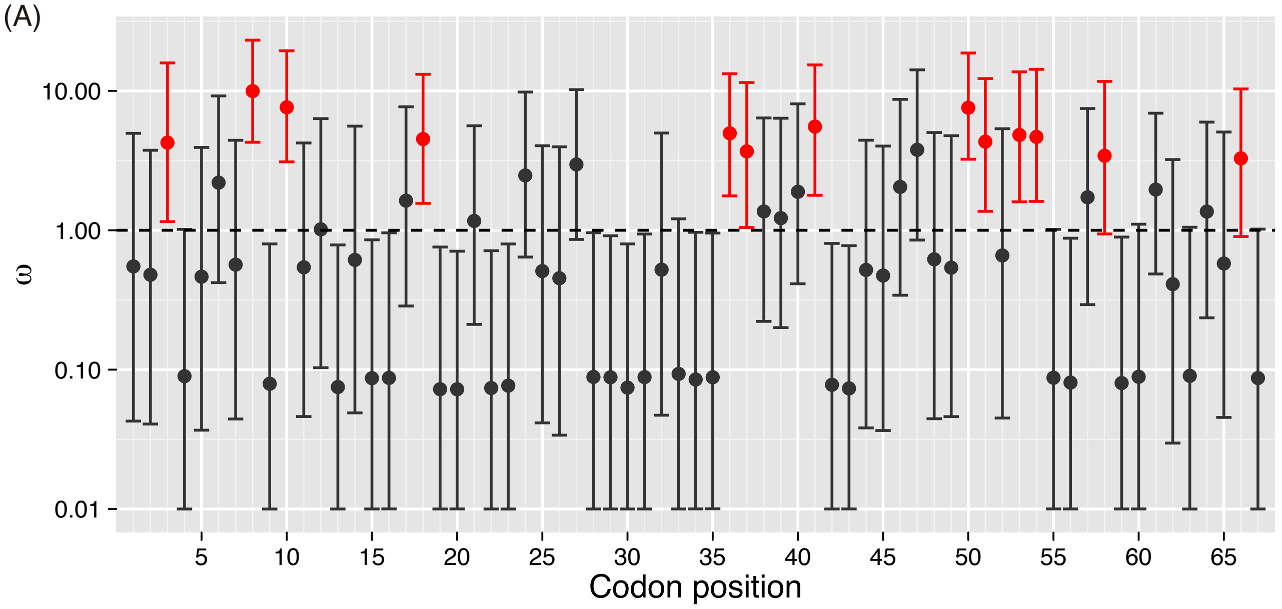

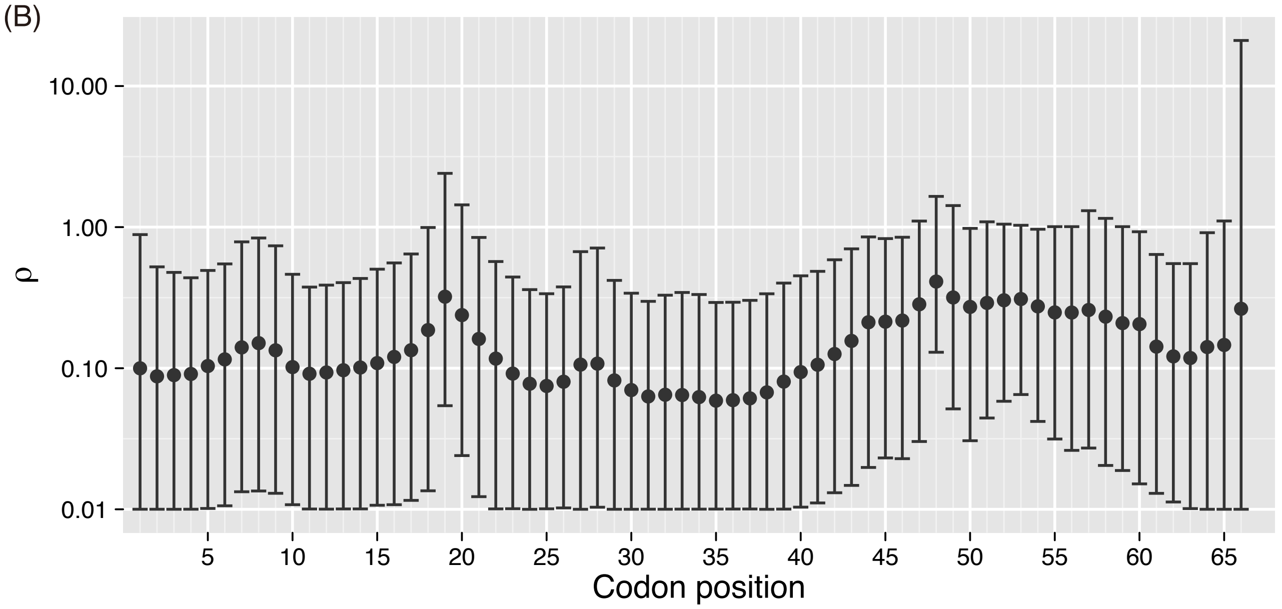

Figure S5 Variation in the d_N/d_S ratio (= ω) (A) and recombination rate (= ρ) (B) along the MHC class IIB exon 2 of the fish owl, generated with omegaMap. Error bars indicate 95% highest posterior density intervals. Codon positions indicated to be under balancing selection (the posterior probability for ω > 1 exceeds 0.95) are colored red.
